# West Nile Fever Outbreak in Horses and Humans, Spain, 2010

**DOI:** 10.3201/eid1712.110651

**Published:** 2011-12

**Authors:** Ignacio García-Bocanegra, Juan A. Jaén-Téllez, Sebastián Napp, Antonio Arenas-Montes, Manuel Fernández-Morente, Vicente Fernández-Molera, Antonio Arenas

**Affiliations:** University of Córdoba, Córdoba, Spain (I. García-Bocanegra, A. Arenas-Montes, A. Arenas);; Consejería de Agricultura y Pesca, Sevilla, Spain (J.A. Jaén-Téllez, M. Fernández-Morente, V. Fernández-Molera);; and Centre de Recerca en Sanitat Animal, Barcelona, Spain (S. Napp)

**Keywords:** West Nile virus, West Nile fever, vector-borne infections, epidemiology, epidemic, horses, humans, viruses, Spain, *Suggested citation for this article*: García-Bocanegra I, Jaén-Téllez JA., Napp S, Arenas-Montes A, Fernández-Morente M, Fernández-Molera V, et al. West Nile fever outbreak in horses and humans, Spain, 2010 [letter]. Emerg Infect Dis [serial on the Internet]. 2011 Dec [*date cited*]. http://dx.doi.org/10.3201/eid1712.110651

**To the Editor:** *West Nile virus* (WNV) is a member of the genus *Flavivirus* within the Japanese encephalitis antigenic complex. The enzootic virus cycle involves transmission between avian hosts and ornithophilic mosquitoes, whereas humans and horses are considered dead-end hosts. Given the recent increase of WNV infection in humans and horses in Europe, concern has been raised regarding public and animal health.

In Spain, WNV seropositivity has been reported for humans (2001), horses (2005–2008), and wild birds (2007–2008) (*1**–**3*). Clinical disease has been described for humans (2004) and raptors (2001–2005) (*4**,**5*) but not for horses. We report the main epidemiologic and clinical findings of a WNV outbreak in horses and humans in Spain in 2010.

After the first clinical case of West Nile fever was detected in a horse in September 2010 in Andalusia (southern Spain), a control program for WNV was initiated that included symptomatic treatment of animals, protection of horses in shelters during hours of higher vector activity, vaccination (not mandatory), vector control using pyrethroid-based insecticides, and elimination of mosquito breeding habitats. Horses with neurologic signs were confirmed as WNV positive by detection of serum IgM against WNV by using a competitive ELISA (IDEXX IgM WNV Ab; IDEXX Laboratories, Westbrook, ME, USA). To assess level of WNV infection within affected herds, samples from sick and clinically healthy unvaccinated horses were collected 2 months after the last case. Serum was tested for IgG against WNV by using a blocking ELISA (Ingezim West Nile compac R.10.WNV.K3; Ingenasa, Madrid, Spain). Positivity was confirmed by a serum microneutralization test (SNT) against WNV (strain Eg101) according to World Organisation for Animal Health guidelines. Blood and cerebrospinal fluid samples from clinically affected horses were analyzed by real-time reverse transcription PCR (*6*).

IgM against WNV was detected in 51 (50%) of 102 clinically ill horses; 15 died and 3 were euthanized. The most common clinical signs were ataxia, disorientation, and weakness, followed by fever, muscular tremor, cranial nerves deficit, and photophobia. Of the 36 infected herds, 30 were located in the province of Cádiz, 5 in Seville, and 1 in Málaga (Figure). The first WNV case was reported on September 10, 2010; the number of cases peaked at 17 in mid-September, then decreased until the last case reported on December 15, 2010. In the second survey, IgG seroprevalence within the 36 infected herds was 51.7% (46/89). All IgM-positive horses and 23 (34%) of 68 clinically healthy horses had antibodies against WNV by blocking ELISA and SNT, indicating intense local transmission in 2010, which contrasts with previous observations (*2*).

**Figure:**
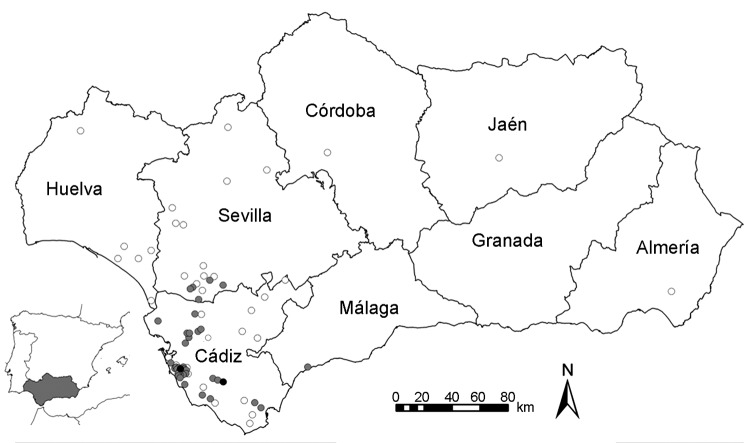
Spatial distribution of West Nile virus–infected horse herds (gray dots), virus-negative horse herds (white dots), and human cases (black dots) in Andalusia (southern Spain) at the end of 2010.

On September 20, 2010, the first case of WNV infection in a 60-year-old man was confirmed by detection of IgM by competitive ELISA and SNT. On October 6, 2010, a 77-year-old case-patient was reported. Both patients, detected in the same area and period of the WNV outbreak (Figure), showed signs of encephalitis but were discharged after several days’ hospitalization. After the human cases were confirmed, control measures, such as inclusion of West Nile fever in the differential diagnosis of neurologic diseases and the control of blood samples from suspected cases and donors, were implemented.

WNV lineage 1 RNA was detected by real-time reverse transcription PCR in blood and cerebrospinal fluid of 1 of the 51 horses analyzed. Despite evidence that WNV persisted during the winter and reemerged during spring in the western Mediterranean, the Spanish strain (JF719069-Spain/10/H-1b) and the strains isolated in Spain in 2007 and 2008 belong to clade 1a and clade 2, respectively (*7*). The closest relatives of the Spain/2010 strain are the 2008 and 2009 Italian strains, with which it seems to share a common ancestor (*7*). Therefore, WNV may have circulated silently in the western Mediterranean region, establishing an endemic cycle after a single introduction. Alternatively, because Andalusia is located within the migratory flyways for wild birds between Europe and Africa, the Spain/2010 strain might have been introduced putatively from Africa from the same source as the Italian strains. Further studies are needed to elucidate the origin of the Spain/2010 strain.

Previous serologic surveys in migratory and resident wild birds from the affected area indicated WNV circulation during 2007 and 2008 (*3*). Although high numbers of dead birds were reported in resident wild birds concurrently with the equine WNF outbreak, it appeared to be caused by another flavivirus, Bagaza virus, not previously found in Europe (*8*). In 2010, Andalusia had the highest rainfall during spring and the hottest summer in the past decade (*9*), which provided optimal conditions for *Culex* spp. mosquitoes. An entomologic survey in the affected area in 2010 showed that the most abundant species was *Cx. pipiens*, with maximum abundance during June and September. The abundance of competent vectors and the high number of wild bird nesting areas in Andalusia provide ideal conditions for the maintenance and circulation of WNV. Therefore, the risk for reemergence of WNV in Spain should be considered high. To improve the early detection of WNV cases and prevent new outbreaks, a surveillance program of passive surveillance in humans, equids, and wild birds; serosurveillance in sentinel horses and wild birds; and entomologic surveillance was initiated after the 2010 outbreak (*10*).
